# Perspective-Taking Increases Willingness to Engage in Intergroup Contact

**DOI:** 10.1371/journal.pone.0085681

**Published:** 2014-01-22

**Authors:** Cynthia S. Wang, Tai Kenneth, Gillian Ku, Adam D. Galinsky

**Affiliations:** 1 Oklahoma State University, Stillwater, Oklahoma, United States of America; 2 Singapore Management University, Singapore, Singapore; 3 London Business School, London, United Kingdom; 4 Columbia University, New York, New York, United States of America; University of Udine, Italy

## Abstract

The current research explored whether perspective-taking increases willingness to engage in contact with stereotyped outgroup members. Across three studies, we find that perspective-taking increases willingness to engage in contact with negatively-stereotyped targets. In Study 1, perspective-takers sat closer to, whereas stereotype suppressors sat further from, a hooligan compared to control participants. In Study 2, individual differences in perspective-taking tendencies predicted individuals' willingness to engage in contact with a hooligan, having effects above and beyond those of empathic concern. Finally, Study 3 demonstrated that perspective-taking's effects on intergroup contact extend to the target's group (i.e., another homeless man), but not to other outgroups (i.e., a man of African descent). Consistent with other perspective-taking research, our findings show that perspective-taking facilitates the creation of social bonds by increasing contact with stereotyped outgroup members.

## Introduction

From local communities to international relationships, a key to building effective interactions with stereotyped outgroups is finding ways to overcome prejudice and stereotyping. One method to decrease stereotyping and prejudice is increasing contact between different social groups [1]. A problem with this method is that although prejudice and stereotyping may beget a host of negative approach behaviors such as out-group derogation, aggression, and even genocide and war [2]–[4], an arguably more basic and common reaction in day to day living is sheer avoidance and a reluctance to have contact with stereotyped outgroups [2], [5], [6]. Thus, although contact can decrease prejudice and stereotyping, prejudice itself may *prevent* willingness to engage in contact. This dilemma raises the question of how to increase willing intergroup contact. In the current research, we suggest that perspective-taking – actively imagining another's viewpoint – will increase individuals' willingness to engage in contact with stereotyped outgroup members.

### Fostering Intergroup Contact

Intergroup contact has numerous social benefits, including reducing prejudice [1], [7]–[10] and anxiety about future intergroup interactions [11]–[13]. These positive effects of contact highlight the importance of establishing conditions that increase individuals' willingness to engage in intergroup contact.

It is, however, difficult to generate willing and positive contact [14]. For instance, although classic research has suggested that eliminating societal and structural constraints (e.g., education and housing differences) can provide the opportunity for contact [15], [16], in reality, group members still remain reluctant to engage with outgroup members [17]. In addition, bringing people into contact when intergroup suspicion is rampant can exacerbate rather than ameliorate prejudice and conflict [18]. Thus, one wants to increase contact but only when people are in a mindset that it will not ironically intensify prejudice.

Over the past 20 years, researchers have investigated various social strategies to diminish prejudice and increase willing contact. One intuitively-appealing strategy for navigating one's diverse social world is to suppress prejudicial thoughts. Although suppression can allow individuals to momentarily decrease the expression of prejudicial thought, post-suppression rebound effects ironically increase both the activation of prejudicial thoughts and avoidance behavior [19]–[22]. For instance, one study found that stereotype suppressors sat further away from a stereotyped target compared to control participants [20].

Other research has found more effective strategies than suppression for producing positive and willing intergroup contact. For example, making individuals interdependent in achieving their goals (e.g., learning from each other to perform well on a test) leads to greater willingness for future interaction [23]. Similarly, focusing on one's emotional reactions when witnessing explicit discrimination predicts willingness to have intergroup contact [24], [25]. More recently, researchers demonstrated that a barrier to initiating contact included fears that intergroup interactions will go poorly [26]. One can overcome these default expectations by focusing on similarities with an outgroup target, which helps to reduce negative interaction expectations [26] and increase individuals' willingness to engage in future interaction [27].

In the current research, we focus on a social strategy that has been argued to effectively create and maintain social bonds – perspective-taking [28]. However, little research on perspective-taking has examined whether perspective-taking actually increases individuals' willingness to have contact with stereotyped targets.

### Perspective-taking

Research has documented perspective-taking's numerous social benefits, including reducing stereotyping, prejudice, and intergroup bias towards the target and the target's group [19], [29]–[32]. Additionally, perspective-taking increases liking [33], satisfaction with interactions [34], [35] and even behavioral coordination [36], [37]. Finally, recent research has shown that perspective-taking can also create approach-oriented actions with perspective-takers willing to sit more closely to a stereotyped-target [38].

Because of these numerous benefits, Galinsky et al. have proposed that perspective-taking is an approach-oriented strategy that is geared towards creating and maintaining social bonds [28]. However, despite this claim, little research has examined whether perspective-taking actually *creates* social bonds (see Todd et al. for an exception [38]). Perspective-taking may result in increased willingness to engage in intergroup contact for two reasons. First, Galinsky et al. suggest that perspective-taking's effects result from a cognitive merging of self and other mental representations [28]. During perspective-taking, the self is applied to the other and this self-other overlap mediates decreased stereotyping [19] and increased helping [39]. Since perspective-takers see more of themselves in others, they should be more likely to approach those individuals. Second, perspective-taking reduces negative, prejudicial evaluations of the target and target group [29]. Since prejudice may create a reluctance to initiate contact [2], [5], decreasing prejudice should increase individuals' willingness to engage in contact. Taking these arguments together, we predict that perspective-taking will increase willingness to engage in contact with stereotyped individuals.

For perspective-taking to be an effective social strategy for stimulating intergroup contact, its benefits should extend beyond the perspective-taking target to the target group more broadly. Indeed, previous research has found that perspective-taking's effects on decreasing stereotyping and prejudice extend from a target to the target's group [19], [29], [40]. Thus, we expected that taking the perspective of a target would lead to a greater willingness to engage in contact with that target's stereotyped group.

Additionally, we explore the boundaries of perspective-taking's effects on willingness to engage in contact. If perspective-taking is a social strategy geared towards building and maintaining specific social bonds [28], its effects may be group-specific and may not generalize to other stereotyped groups. Consistent with this theorizing, research has shown that perspective-taking effects tend to be group-specific, with perspective-taking decreasing prejudice towards the target group but not towards other groups [31], [32]. For instance, Shih et al. found that after taking the perspective of Asians, perspective-takers felt more empathy towards and liked other Asian targets more, but these effects did not extend to White and African American targets [31]. In another study, Vescio et al. found that after taking the perspective of an African American student, perspective-takers showed more positive attitudes towards African Americans in general, but these effects did not influence attitudes towards other stereotyped groups such as homosexuals [32]. Thus, we predicted that the effects of perspective-taking on intergroup contact would be target group-specific.

### Overview

We conducted three studies to examine whether individuals' willingness to engage in contact with stereotyped targets can be increased through perspective-taking. We manipulated perspective-taking in Studies 1 and 3, and measured perspective-taking tendencies in Study 2. In Study 1, we explored whether perspective-takers would actually sit closer to a stereotyped target compared to participants in two control conditions and a stereotype suppression condition. In Study 2, we examined whether perspective-taking tendencies would predict willingness to engage in contact with a stereotyped target. Finally, in Study 3, we considered whether taking the perspective of a stereotyped target would increase willingness to engage in contact with individuals from a range of stereotyped groups or only with the target's group.

Overall, we sought to replicate existing findings [20], [31], [38], but also to provide new and important insights. First, by including perspective-taking, stereotype suppression, and two control conditions in Study 1, as well as targets from the same and different outgroups in Study 3, we hoped to provide a comprehensive understanding of the effectiveness of different social strategies for facilitating intergroup contact. Second, in contrast to most other perspective-taking studies, two of our studies examined our hypotheses in Singapore, an Eastern culture, allowing us to understand the robustness of perspective-taking effects. All study materials and data are available from the authors upon request.

## Study 1

Study 1 tested whether perspective-takers would be willing to make physical contact with a stereotyped target by sitting closer to that person. In addition, we wanted to provide a more comprehensive understanding of different social strategies used to navigate the multicultural landscape. As mentioned earlier, although an intuitively-appealing alternative for dealing with diverse outgroups is stereotype suppression, past research has shown that suppressing stereotypical thoughts can result in avoidance rather than approach behavior. For instance, in Macrae et al. 's study, contact was impeded by stereotype suppression, with suppressors sitting further away from the target than did control participants [20].

In Study 1, people wrote a narrative essay about a person in a photograph – a person who represented the outgroup of hooligans. We manipulated the essay writing instructions to create a perspective-taking condition, a suppression condition, and two control conditions; one of the control conditions induced an objective focus [41] and the second control condition did not provide instructions for how to write the essay [19], [20]. Next, we told participants that they would meet with the person in the photograph [20]. We measured how close participants sat to where the target was allegedly sitting. Overall, we predicted that because perspective-taking is approach-based and suppression is avoidance-based [28], [42], perspective-takers would sit closer to the target than the two sets of control participants (consistent with Todd et al. 's findings [38]). We also expected that suppressors would sit further away from the stereotyped target than would participants in the two control conditions (consistent with Macrae et al. 's findings [20]). Given that previous research has found similar effects with different types of control conditions [37], we did not expect any difference in seating distance between the two control conditions.

### Method

#### Pretest

We used a target that was meaningful for our Singaporean participants – an “Ah Beng” or local hooligan. To pretest whether Ah Bengs are seen as negative stereotypes, 17 participants rated whether a number of traits were typical of that group on a 7-point scale (1 =  *not at all relevant to Ah Bengs* and 7 =  *very relevant to Ah Bengs*). Traits were seen as stereotypical if they were rated significantly above the scale's midpoint [19]. In addition, participants indicated how favorable society's view is of Ah Bengs on a 7-point scale (1 =  *not at all favorable* and 7 =  *very favorable*). If Ah Bengs were rated significantly below the scale's midpoint, they were considered as having an overall negative stereotype.

The pretest revealed that Ah Bengs were seen as stereotypically aggressive, crude, and reckless, *t*(16) 's >8.28, *p's* <.001, traits that would naturally lead to avoidance and less willingness to engage in contact. Overall, Ah Bengs were viewed negatively, *t*(16)  = 6.34, *p*<.001.

#### Participants and Design

Participants were 116 undergraduate students (57 men and 59 women) from the National University of Singapore (NUS) who received course credit for participation. The study had four between-participants conditions: perspective-taking vs. suppression vs. objective vs. control. This study received approval from the NUS Institutional Review Board #NUS-1151 (http://www.nus.edu.sg/irb/) and participants provided written consent before beginning the experiment.

#### Procedure

Participants were shown a photograph of a person and asked to write about a typical day in that person's life. The photograph was of an Ah Beng, a young Asian male with spiky hair and tattoos. In the *perspective-taking* condition, participants were instructed, “Take the perspective of the individual in the photograph and imagine a day in the life of this individual as if you were that person, looking at the world through his eyes and walking through the world in his shoes” [19]. In the *suppression* condition, participants read, “Previous research has noted that our impressions and evaluations of others are consistently biased by stereotypic preconceptions. When constructing the passage you should actively try to avoid thinking about the photographed individual in such a manner” [20]. In the *objective* condition, participants were instructed, “Try to be as objective as possible when imagining what is happening to this person and what his day is like. Try not to let yourself get caught up in imagining what this person has been through or how the person feels. Just describe the person as objectively as possible” [41]. In the *control* condition, participants read “Please compose a brief passage describing a typical day in the life of the individual in the photograph” [20].

#### Seating distance

After completing the essay, participants were given an opportunity to meet the photographed person. Participants entered another room and saw a row of eight empty seats with a helmet on the first seat. While waiting for the target's return, the participant was asked to take a seat. The seat that the participant chose to occupy was the dependent variable (seat 2–8).

The experimenter left the room for a few minutes and upon returning remarked that she could not find the target. Participants then filled out the final demographic questionnaire and were debriefed about the purpose of the study. None of the participants expressed any suspicion.

### Results

**Figure 1:**
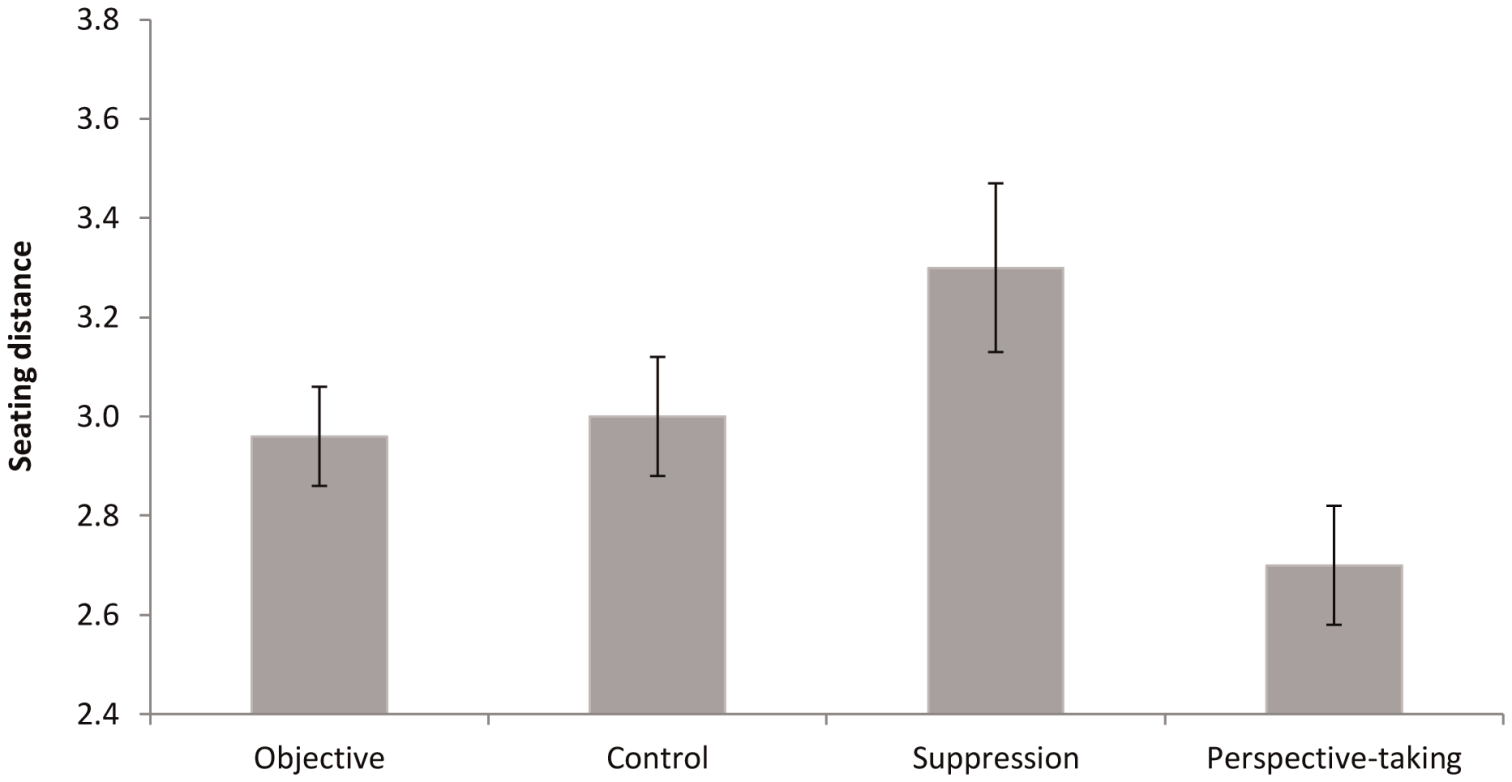
Effect of the narrative essay writing instructions on seating distance in Study 1.

We conducted a single factor (narrative essay instructions: perspective taking vs. suppression vs. objective vs. control) ANOVA on participants' seating distance. As expected, there was a significant effect of narrative essay instructions, *F*(3, 112)  = 3.58, *p*<.02 (Figure 1).

Perspective-takers (*M* = 2.70, *SD* = .65) sat closer to the Ah Beng than did suppressors (*M* = 3.30, *SD* = .87), *t*(112) = 3.27, *p* = .001, *d* = .78, and marginally closer compared to control (*M* = 3.00, *SD* = .68) and objective (*M* = 2.96, *SD* = .51) participants, *t*(112) = 1.83, *p* = .07, *d* = .45. Suppressors sat further away from the Ah Beng than did the control and objective participants, *t*(112)  = 1.97, *p* = .05, *d* = .42. There were no differences in seating distance between participants in the objective and control conditions, *t*(112)  = .20, *p* = .84, *d* = .07. Overall, perspective-taking increased and suppression decreased participants' willingness to have physical contact with a stereotyped target.

## Study 2

Whereas Study 1 manipulated perspective-taking, Study 2 measured perspective-taking tendencies. By doing so, we examined the effects of natural variations in perspective-taking tendencies on participants' willingness to engage in contact with the target. We also measured empathic concern since perspective-taking and empathy are often studied together [35], [36], [43] and are seen as interrelated constructs [40], [43]. Despite their seeming similarities, past research has found that individual differences in perspective-taking tendencies are better predictors of reduced stereotyping [44] and more effective interpersonal interactions in the form of mimicry and negotiation outcomes [35], [36] than are individual differences in empathic concern. Given our interests in understanding perspective-taking's effects, we hypothesized that perspective-taking tendencies would predict intergroup contact, even after controlling for the effects of empathic concern. Study 2 also used a different form of approach than Study 1. We asked participants if they would be willing to interact with the target in the future.

### Method

#### Participants and Design

Thirty-one undergraduate students (14 men and 17 women) from NUS participated for course credit. Perspective-taking tendency and empathic tendency were the main predictor variables. This study received approval from the NUS Institutional Review Board #NUS-1151 (http://www.nus.edu.sg/irb/) and participants provided written consent before beginning the experiment.

#### Procedure

Participants were shown the photograph of the Ah Beng used in Study 1 and were asked to write an essay about his typical day. All participants were asked to write the narrative essay using the *control* condition instructions from Study 1. By using these neutral instructions, we could test the effects of natural variations in perspective-taking and empathic tendencies on intergroup contact [35], [44]. Participants were next informed that they would meet the photographed individual.

#### Perspective-taking tendencies and empathic tendencies

While they were waiting, participants completed Interpersonal Reactivity Index [43] on 5-point scales (0 =  *Does not describe me well* and 4 =  *Describes me very well*) which included a 7-item perspective-taking subscale (e.g., “I sometimes try to understand my friends better by imagining how things look from their perspective”) and a 7-item empathy subscale (e.g., “I often have tender, concerned feelings for people less fortunate than me”).

#### Willingness to engage in contact

The experimenter went to look for the person and after a few minutes returned and told participants that they could not find him. The experimenter, who was blind to the hypothesis, asked participants, “Since you didn't get a chance to interact with the person today, we would like to arrange another day for you to meet with him to exchange life experiences. Would you be willing to meet up with him?” Our dependent measure was whether (“yes” or “no”) participants agreed to meet with the target.

Participants were probed for suspicion before being debriefed and dismissed. None expressed suspicion.

### Results

Table 1 presents descriptive statistics, Cronbach's *α*'s, and bivariate correlations for the variables. We performed a logistic regression analysis with willingness to engage in intergroup contact (“yes” or “no”) as the dependent variable. Overall, 15 participants (48.4%) agreed to meet with the target. As can be seen in Table 2, perspective-taking tendencies predicted participants' willingness to engage in contact with the Ah Beng (Model 1). When both perspective-taking tendencies and empathic tendencies were entered simultaneously (Model 2), perspective-taking tendencies still predicted willingness to have future contact with the Ah Beng. In our final model, age and gender were included as control variables; neither age nor gender were associated with willingness to engage in contact, but perspective-taking tendencies still predicted intergroup contact.

**Table 1:** Descriptive Statistics, Cronbach's *α*, and Bivariate Correlations for Study 2 Variables.

Variable	No. of items	M	SD	α	1	2	3	4	5
1. Perspective-taking tendencies	7	2.45	.66	.79	-				
2. Empathic tendencies	7	2.68	.57	.69	.13	-			
3. Gender	-	-	-	-	.06	−.28	-		
4. Age	-	20.45	2.08	-	.05	.23	.24	-	
5. Willingness to engage in contact	-	-	-	-	.41*	−.03	.03	.07	-

*Note*: Gender was coded as 0 =  female and 1 =  male. Willingness to engage in contact was coded as 0 =  no and 1 =  yes.

*p≤.05

**Table 2:** Stepwise Logistic Regression with Willingness to Engage in Contact as the Dependent Variable in Study 2.

Factors	Model 1	Model 2	Model 3
**Predictors in Models**			
Constant	−3.66	−2.77	−5.01
Perspective-taking tendencies	1.46 (.69)*	1.52 (.71)*	1.61 (.75)*
Empathic tendencies		−.39 (.74)	−.66 (.91)
Gender (0 = female; 1 = male)			−.37 (.97)
Age			.14 (.24)
**Model Performance**			
R^2^	.17	.17	.18

*Note*: The entries are unstandardized coefficient estimates with standard errors in parentheses. Willingness to engage in contact was coded as 0 =  no and 1 =  yes.

*p≤.05

Thus, perspective-taking tendencies were associated with greater willingness to engage in contact with the Ah Beng, having effects above and beyond those of empathy. Perspective-taking tendencies predicted willingness to engage in contact even though all participants were exposed to the same stereotype and given the same control instructions.

## Study 3

Although perspective-taking increased willingness to engage in contact with an Ah Beng in Studies 1 and 2, several questions remain that we address in Study 3. First, we considered whether our perspective-taking effects extend to a different member of the same target group. Consistent with previous findings that perspective-taking's effects on decreasing stereotyping and prejudice extend from a target to the target's group [19], [29], [40], we predicted that taking the perspective of a homeless man would lead to a greater willingness to engage in contact with another homeless man. Second, we considered whether taking the perspective of one stereotyped group would increase willingness to engage in contact with other stereotyped groups. Because research by Shih et al. and Vescio et al. has shown that the effects of perspective-taking tend to be group-specific [31], [32], we predicted that, compared to control participants, perspective-takers would be more willing to engage in contact with a member of the same target group (another homeless man), but not with a member of a different target group (man of African descent).

Finally, to further examine the robustness of our effects, we consider a new stereotyped target – the homeless and performed this study in a different country – the United Kingdom.

### Method

#### Pretest

Using the same pretest procedure as in Study 1, 14 participants reported that the homeless are seen as stereotypically aggressive, dirty, disruptive, lazy, needy, and sickly, *t*(13) 's >2.14, *p's* <. 05, traits that would lead to less willingness to engage in contact. Overall, the homeless are viewed negatively, *t*(13)  = 6.20, *p*<.001.

#### Participants and Design

Participants were 148 individuals (56 men and 92 women) recruited from the London Business School (LBS) participant pool who received £10 for participation. We only included individuals who reported English as a first language because the participant pool includes individuals from numerous countries where English is not the native language and because our manipulation and task require fluency in English. This left 112 individuals (43 men and 69 women). Study 3 had a 2 (narrative essay instructions: perspective-taking vs. control) X 2 (target group: same vs. different) between-participants design. This study received approval from the LBS Institutional Review Board in January 2010 and participants provided written consent before beginning the experiment.

#### Procedure

Participants were shown a photograph of a homeless man who was unkempt and lying next to a garbage can, and were asked to write about a day in his life. Perspective-taking and control instructions were the same as in Study 1.

After completing the narrative essay, participants were shown a photograph of a different individual. In the *same-target-group condition*, participants were shown a photograph of another homeless man. In the *different-target-group condition*, participants were shown a photograph of a man of African descent. Participants were told that the photographed person had agreed to participate in a separate study, which required two people to interact and work together. Participants were asked whether they would be willing to participate in the study with this person (yes or no) and the number of tasks (1–6) that they would be willing to engage in with the person (participants were informed that if they chose to complete fewer than 6 tasks with the photographed individual, there would be alternate tasks for them such that the total time commitment was the same). We combined these two measures into a single dependent measure of the number of tasks participants were willing to engage in with the target (0–6 tasks).

### Results

We predicted that, compared to control participants, perspective-takers would be more willing to engage in contact with a member of the same target group (i.e., another homeless man), but not with a member of a different target group (i.e., man of African descent).

**Figure 2:**
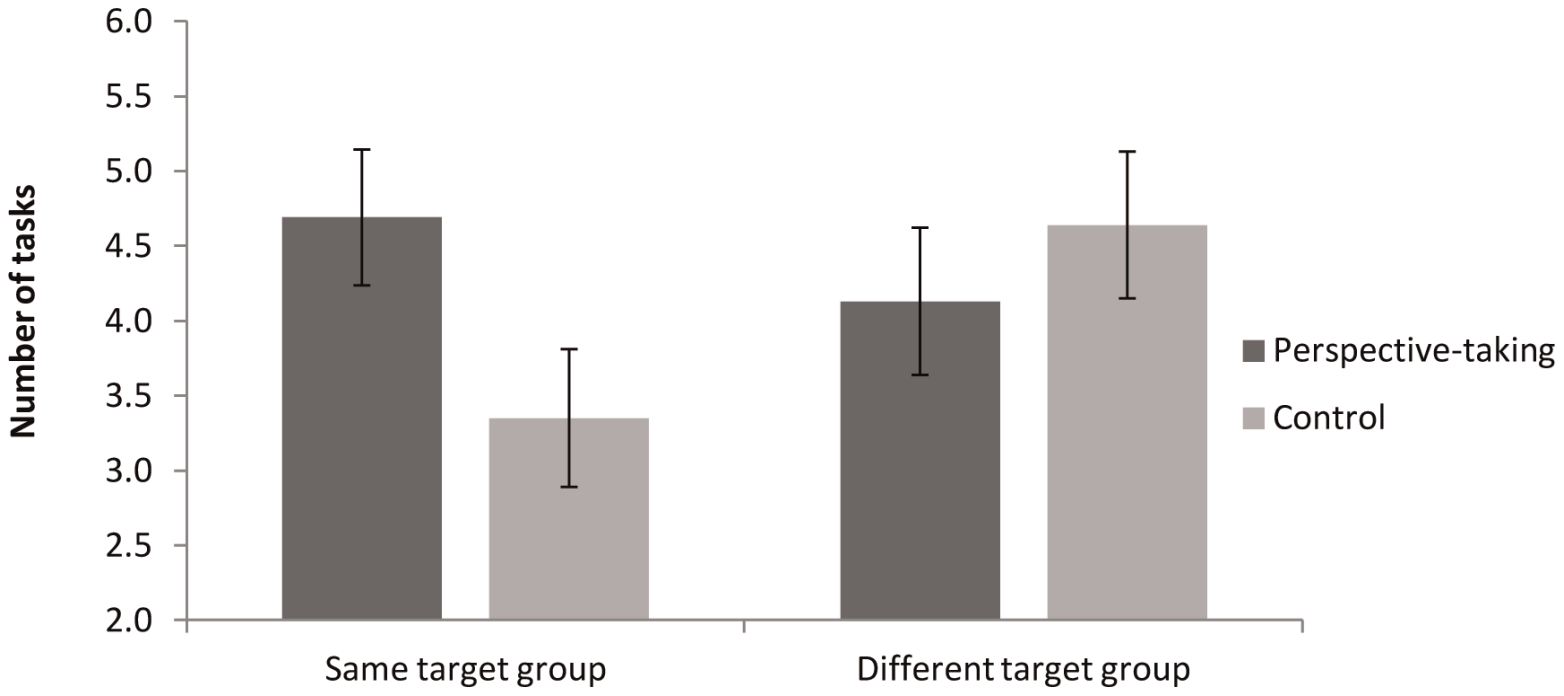
Effects of writing instructions and target group on number of volunteered tasks in Study 3.

The 2 (narrative essay instructions) X 2 (target group) between-participants ANOVA on the number of tasks participants were willing to engage in revealed a significant interaction, *F*(1, 108)  = 3.94, *p* = .05 (Figure 2). Contrast analyses showed that, in the same-target-group condition, perspective-takers chose to participate in more tasks with the homeless man (*M* = 4.69, *SD* = 2.31) compared to control participants (*M* = 3.35, *SD* = 2.74), *t*(108) = 2.05, *p* = .04, *d* = .53. For the different-target-group condition, there was no difference in the number of tasks perspective-takers (*M* = 4.13, *SD* = 2.43) and control participants (*M* = 4.64, *SD* = 2.22) were willing to engage in with the target, *t*(108)  = .76, *p* = .45, *d* = .14.

Consistent with theorizing [28] and empirical work on prejudice [31], [32] showing that perspective-taking is a social strategy geared towards the formation and maintenance of specific social bonds, the current results show that perspective-takers were more willing to engage in contact with another member of the same target group; however, these benefits do not generalize to other target groups.

## General Discussion

Across three studies, we found that perspective-taking increased individuals' willingness to engage in contact with stereotyped outgroup members. In Study 1, we manipulated perspective-taking and found that perspective-takers sat closer to a hooligan. In contrast, stereotype suppression resulted in participants sitting further away from the target. In Study 2, we measured perspective-taking tendencies and found that it predicted willingness to engage in contact with a hooligan. Finally, Study 3 showed that perspective-taking's effects on intergroup contact extend to other members of the perspective-taking target's group, but not to other stereotyped groups.

When people are not affected by intergroup suspicion and enmity [18], intergroup contact sets the stage for decreased prejudice and for the development of long-term relationships [1]. However, attempts at increasing positive and willing intergroup contact are not always successful [20]. Given the difficulties of increasing individuals' willingness for intergroup contact, particularly with negatively-stereotyped targets, the current research considered a social strategy – perspective-taking – that we hypothesized would increase willing and positive intergroup contact. We focused on perspective-taking because it is a social strategy that is geared towards approach tendencies as well as building and maintaining social bonds [28], [42]. As predicted, we found that perspective-taking increased individuals' willingness to engage in contact with stereotyped outgroup members.

The current research replicates existing findings [20], [31], [32], [38] but also provides new and important insights. First, Studies 1 and 3 contribute to our understanding of the effectiveness of different social strategies by providing a more comprehensive test, pitting perspective-taking against suppression and two control conditions (Study 1) and including targets from the same and different stereotyped groups (Study 3). At a basic level, not all social strategies are alike – although stereotype suppression seems like an intuitively-appealing strategy when dealing with outgroup targets, our findings and those of Macrae et al. [20] highlight that good intentions to suppress one's stereotypes can ironically result in avoidance rather than approach. Importantly, Study 1 showed that the only social strategy that increased contact was perspective-taking; the control and objective participants were less willing to approach the Ah Beng than perspective-takers were.

Theoretically, our finding that being objective did not increase intergroup contact is reminiscent of the effects of multiculturalism versus color-blindness on racial bias and intergroup interaction [45]–[47]. For example, a colorblind approach led to worse intergroup interactions than did multiculturalism [46]. Trying to be objective or colorblind is not as effective for increasing contact and decreasing prejudice as trying to take the other's perspective and appreciating the richness of one's multicultural world.

Findings from Study 3 blend together our core interest of perspective-taking induced intergroup contact with existing work on the generalizability of perspective-taking's effects [31], [32]. We found that perspective-takers were willing to interact with another member of the stereotyped target's group but not with another stereotyped group. This finding demonstrates that perspective-taking is a social strategy geared towards building and maintaining *specific* social bonds, and not just any bonds.

To further explore our data, in Studies 1 and 3, we coded participants' narrative essays for the amount of stereotyping of the Ah Beng (Study 1) and homeless man (Study 3). Using Monteith, Spicer, and Tooman's (1998) coding system, one of the authors and a research assistant, both blind to condition, first examined the passages and generated an exhaustive list of stereotypes that appeared in the essays (e.g., An Ah Beng is a gang member and drinks alcohol). Next, two coders parsed the passages into thought units (i.e., any complete thought) and coded each unit according to whether it reflected one of the stereotypes. Essay stereotypicality was operationalized as the number of thought units with stereotypic content as a proportion of total thought units. Inter-rater reliability was high for both studies, *r*'s ≥.72, *p*'s<.001.

For Study 1, essays were only available for 78 of the 116 participants (the others were lost because of various office moves). A single factor (narrative essay instructions: perspective taking vs. suppression vs. objective vs. control) ANOVA on essay stereotypicality was significant, *F*(3, 74)  = 4.47, *p* = .006. The stereotypicality of perspective-takers' essays (*M* = .47, *SD* = .25) did not differ from the essays in the two control conditions (*M* = .37, *SD* = .27 and *M* = .52, *SD* = .34 for objective and control conditions respectively), *t*(74)  = .38, *p* = .70, *d* = .09. Stereotype suppressors (*M* = .22, *SD* = .22) wrote essays that contained less stereotypicality than the essays of perspective-takers, *t*(74)  = 2.83, *p* = .006, *d* = 1.06, and control and objective participants, *t*(74) = 2.86, *p* = .006, *d* = .66. For Study 3, perspective-takers (*M* = .49, *SD* = .25) wrote less stereotypical essays than did control participants (*M* = .61, *SD* = .30), *t*(110)  = 2.29, *p*<.03, *d* = .44.

Overall, the essay coding findings from Study 1 contradict those from Study 3 and those from Galinsky and Moskowitz (2000), who found that both stereotype suppressors and perspective-takers expressed less stereotyping in their essays than did control participants. Given the reduced sample size in Study 1 and the inconsistent findings between the two studies, it is difficult to conclude the effects of perspective-taking on essay stereotypicality, let alone whether essay stereotypicality has meaningful downstream consequences on attitudes and behaviors. Further research should examine these issues as it could provide interesting theoretical and practical insight into perspective-taking.

Another important contribution from our research is the use of an East-Asian context. Although most perspective-taking studies have been conducted in Western countries, two of our three studies were conducted in Singapore, an Eastern culture. Although we did not have specific hypotheses about how culture might moderate the effects of perspective-taking on intergroup contact, a replication of Todd et al. 's [38] perspective-taking induced approach findings is not trivial since people in Eastern and Western cultures have different conceptualizations of the self. Specifically, whereas Easterners have a more interdependent, collectivistic sense of the self, Westerners tend to see the self as more independent [48]. Given this difference in how the self is viewed and also the fundamental role of the self in perspective-taking [19], [28], it is theoretically and practically noteworthy to see that perspective-taking increased intergroup contact in our Singaporean participants.

Research exists by Vorauer and her colleagues that contradict the current findings and those of Todd et al. [38], showing that perspective-taking and empathy manipulations can increase evaluative concerns and lead to more negative intergroup interactions [49], [50]. There are a number of notable differences between the combination of the current research and Todd et al. 's studies and those of Vorauer and her colleagues. First, Vorauer and her colleagues do not show that perspective-taking has a main negative effect on intergroup interaction. Rather, they demonstrate a statistical interaction with initial levels of prejudice – perspective-taking does not backfire in general, but only for low-prejudiced individuals [49]. Second, the procedures differ across the streams of research. In Vorauer and her colleagues' studies, a) the perspective-taking/empathy manipulations occur *after* participants are aware of the upcoming intergroup interaction and b) the intergroup interaction centers around the experiences of the outgroup target. In the present studies and Todd et al. 's studies [38], a) the perspective-taking manipulation took place *before* any awareness of an intergroup interaction and b) the entire paradigm (the manipulations and the interaction) was not explicitly focused on the experiences of the stigmatized individual. Thus, for participants in Vorauer and her colleagues' studies, the knowledge of the upcoming interaction and the focus of that interaction might have influenced the perspective-taking manipulation. In contrast, for Todd et al. 's [38] and our participants, there was no expectation to interact with the target until after the experimental manipulation took place and the focus of the interaction was not infused with intergroup anxiety. Finally, Vorauer and colleagues' research have focused on one specific target group: Aboriginals from Canada. In contrast, the positive effects of perspective-taking on prejudice reduction and intergroup interaction have been shown across a number of groups – African Americans [32], [37], [38], Hispanics [51], the elderly [19], [29], [37], hooligans (the current research), occupational groups [44], and medical patients [34]. Future work should examine social groups other than Aboriginal Canadians to test the generalizability of the findings demonstrating the detrimental role of perspective-taking on prejudice and intergroup interactions.

Overall, these contradictory sets of findings provide fodder for future research to examine when perspective-taking will have a positive vs. negative effect on intergroup interactions. Based on our comparison of the two sets of findings, future research should vary when perspective-taking manipulations occur, the focus of the intergroup interaction, and the stigmatized groups used.

### A Virtuous Cycle

We propose that perspective-taking can encourage a virtuous cycle that promotes stronger bonds with stereotyped outgroups. Intergroup interventions require two features to be successful: they need to decrease prejudicial thoughts and increase intergroup contact. When people are in a prejudicial frame of mind, intergroup contact can actually increase conflict [18]. Fortunately, perspective-taking has been shown to immediately decrease prejudicial thoughts [19], [29], and as we have shown here, increase willingness to engage in contact with stereotyped targets. Thus, perspective-taking simultaneously puts people in a less prejudicial frame of mind and increases intergroup contact. Once positive intergroup contact occurs, prejudice will further decrease [1]. Additionally, perspective-taking also greases the cogs of social interactions by increasing behavioral coordination [30], [36], [37]. Overall, perspective-taking appears to be a critical engine in promoting intergroup harmony, helping to create and maintain long-lasting positive social bonds.
